# A peer learning intervention in workplace introduction - managers’ and new graduates’ perspectives

**DOI:** 10.1186/s12912-021-00791-0

**Published:** 2022-01-04

**Authors:** Ylva Pålsson, Maria Engström, Christine Leo Swenne, Gunilla Mårtensson

**Affiliations:** 1 Faculty of Health and Occupational Studies, University of Gävle, Kungsbäcksvägen 47, 801 76 Gävle, Sweden; 2 Department of Public Health and Caring Sciences, Uppsala University, Box 564, 751 22 Uppsala, Sweden

**Keywords:** Collaborative learning, Feasibility, Intervention, Medical Research Council (MRC) framework, Newly graduated nurses, Peer learning, Process evaluation, Randomized controlled trial, Social learning

## Abstract

**Background:**

Evaluation of a complex intervention are often described as being diminished by difficulties regarding acceptability, compliance, delivery of the intervention, recruitment and retention. Research of peer learning for nursing students have found several positive benefits while studies of peer learning for newly graduated nurses are lacking. This study aimed (1) to investigate the study process in terms of (a) first-line managers’ perspectives on the intervention study, the difficulties they face and how they handle these and (b) new graduates’ fidelity to the intervention and (2) to examine the effect of the peer learning intervention in workplace introduction for newly graduated nurses.

**Methods:**

A mixed-methods approach using semi-structured interviews with eight managers, repeated checklist for fidelity and questionnaires conducted with 35 new graduates from June 2015 and January 2018, whereof 21 in the intervention group. The peer learning intervention’s central elements included pairs of new graduates starting their workplace introduction at the same time, working the same shift and sharing responsibility for a group of patients for 3 weeks. The intervention also included 3 months of regular peer reflection.

**Results:**

Managers offered mostly positive descriptions of using peer learning during workplace introduction. The intervention fidelity was generally good. Because of recruitment problems and thereby small sample size, it was difficult to draw conclusions about peer learning effects and, thus, the study hypothesis could either be accepted or rejected. Thereby, the study should be regarded as a pilot.

**Conclusions:**

The present study found positive experiences of, from managers, and fidelity to the peer learning intervention; regarding the experimental design, there were lessons learned.

**Trial registration:**

Before starting data collection, a trial registration was registered at (Trial ID ISRCTN14737280).

**Supplementary Information:**

The online version contains supplementary material available at 10.1186/s12912-021-00791-0.

## Introduction

Transitioning into a new professional role is challenging. While many new graduates adapt effectively to their workplace, others may struggle to manage these demands [1]. Studies have reported that new graduates who have the opportunity to meet, socialize and share experiences assist each other in coping with stress [2]. Furthermore, that having a peer involved sharing and acknowledging each other’s experiences and feelings, asking each other as they say “stupid questions”, and admitting to shortcomings and worries, all of which reduced feelings of stress and anxiety [3]. The present study is part of a research project designed to investigate new graduates’ pathways into the profession. It focuses on the use, feasibility and effects of peer learning. The conclusion from our earlier feasibility study was that the peer learning intervention seemed to be feasible in the study context [3]. The present study is an extension and aims to look at the process and effect of using a peer learning intervention in workplace introduction for new graduates. Boud’s [4] theoretical description of peer learning guided the intervention and Medical Research Council (MRC) framework of complex interventions and the concept of process evaluation [5, 6] were used to guide the study design.

## Background

Peer learning is a pedagogical model that originates from theorists of social learning such as Bandura [7]. It is based on the idea that experience, understanding, and knowledge are built and developed in interactions between humans. According to Boud [4] peers learn from each other’s insights and understanding, reasoning, and actions through communication. To our knowledge, there are no previous studies focused on using peer learning as a deliberate support strategy in new graduates’ transition to work, except of Pålsson et al. [3], whilst studies on nursing students using peer learning during clinical practice are several. Studies mostly shown positive outcomes and advantages, as belief in oneself and increased self-efficacy [8], confidence [9–11], development and clinical knowledge [10, 12, 13], capacities also desirable among new graduates. Students have report that working together and supporting each other reduce stress and anxiety [10, 11]. Furthermore, when they are introduced to staff and face new clinical challenges, they feel safer and less nervous being with a peer [14]. Few limitations associated with peer learning have been described, but students have reported disadvantages, such as having to share scarce resources with the peer [15] and reduced learning when personalities or learning styles clash [10, 16].

The importance of relational aspects in facilitating transition (support from other nurses’ peers, mentors and preceptors) were revealed in a review by van Rooyen et al. [17]. Further, a mixed-methods study [18] demonstrated that self-efficacy, job satisfaction, stress and structural empowerment were factors affecting new graduates’ transition. The facilitators, which supported the quantitative findings, were self-confidence, interaction with colleagues, positive and supportive work environments, and a transition program. According to Spreitzer [19] a supportive peer relationship played an important role in facilitating empowerment. Kuokkanen et al. [20] reported a positive relationship between new graduates’ assessment of empowerment and professional competence, job satisfaction and satisfaction with the quality of care. Furthermore, relational aspects have shown to have positive impact in new graduates’ job satisfaction [21]. A review by Hawkins et al. [22] indicated that new graduates not prepared for situations in which professional and organizational constraints influence their work, results in feelings of unpreparedness that cause lack of confidence as well as feelings of uncertainty, anxiety and stress. Earlier studies found that new graduates (peers) who have had the opportunity to meet, socialize and share experiences in a group were better able to cope with stress [2, 23].

Besides support from other nurses, first-line managers have been reported to be important in new graduates’ transition into the workplace. A qualitative study showed that new graduates and managers identified similar factors that facilitated transition [24]. Managers are important in the process of testing new aspects of new graduates’ introduction. The managers recruit and hire new graduates for the wards and are responsible for their workplace introduction. In an earlier quasi-experimental study, Henderson et al. [25] reported on the importance of first-line managers’ engagement for the success of practice interventions, as first-line managers contextualize projects for their staff.

In the present study, process evaluation and the MRC framework provide guidance for the study design. The MRC framework can be helpful when developing, evaluate and implement complex interventions [5, 6]. The complex intervention of peer learning involves two new graduates undergoing workplace introduction together. The pair of new graduates share a preceptor and are therefore able to support and learn from and with each other as well as with the preceptor and the team. This introduction is different from the traditional one in which one preceptor guides one new graduate. Complex interventions are described as interventions that contain several interacting components, where these components may interact with the context during intervention delivery [6]. The peer learning intervention consisted of several components, and adding peer learning to the existing introduction program affected, e.g., the new graduates, first-line managers, preceptors, and colleagues, such as other nurses and nurse assistants. To evaluate the effect of an intervention, a randomized controlled trial (RCT) design is generally regarded as the gold standard when randomization is feasible. However, evaluations are often described as being diminished by difficulties regarding acceptability, compliance, delivery of the intervention, recruitment and retention [5]. In the updated version of the MRC framework, greater attention to the context in which interventions take place has been added. The aim of a process evaluation is to provide a more detailed understanding of a complex intervention. The findings can help us understand the casual assumptions supporting the intervention and how interventions work in practice [6].

**Fig. 1:**
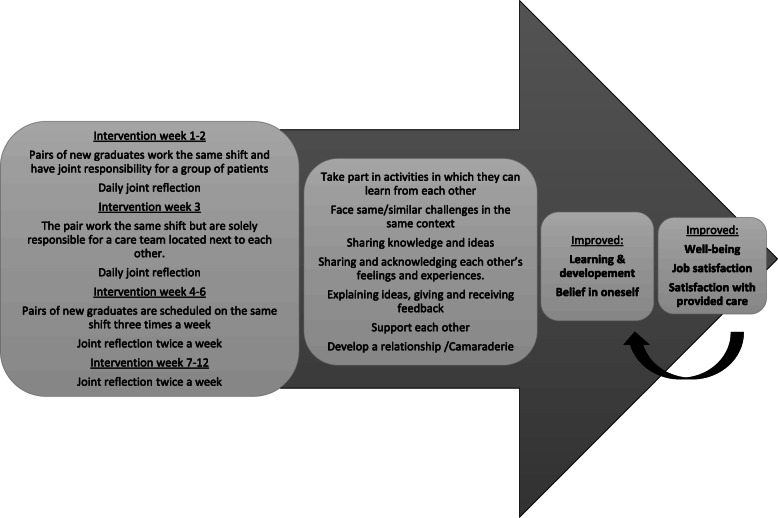
Program logic assumption with measured outcomes shown in bold

To sum up, it is important to support new graduates during the first period so as to build relationships that increase their patient care capacity, confidence, competence and job satisfaction. It seems reasonable to assume that new graduates could achieve the same positive outcomes of peer learning as described by nursing students and an earlier feasibility study support that new graduates’ descriptions of peer learning during their workplace introduction was concordant with the theoretical model [3]. Based on earlier studies, our assumption was that peer learning that includes working and learning together and supporting each other [10, 11] should influence new graduates’ learning and development as well as belief in themselves, thereby increasing their satisfaction and well-being (Fig. 1). However, more research on employing peer learning in the context of workplace introduction is needed, and the present study can contribute to this area. Nevertheless, complex interventions in healthcare are often related to problems such as difficulties with standardizing the design, delivering the intervention and being sensitive to structures of the local context [5]. Thus, to provide a more detailed understanding of the intervention, a process evaluation was conducted alongside the effect evaluation.

## Methods

### Aim

The study aim was twofold: (1) to investigate the study process in terms of (a) first-line managers’ perspectives on the intervention study, the difficulties they face and how they handle these and (b) new graduates’ fidelity to the intervention and (2) to examine the effect of the peer learning intervention in workplace introduction for newly graduated nurses.

We hypothesized that newly graduated nurses who are given the opportunity to learn the profession with a peer would improve significantly more over time regarding self-rated ability to perform nursing-specific tasks and competences (self-efficacy), learning and vitality (thriving) than would new graduates in a control group who were introduced to the profession in the traditional manner. We further hypothesized that the newly graduated nurses exposed to peer learning would perceive greater psychological empowerment, well-being, job satisfaction, satisfaction with given care and less stress/demands.

### Design

The study design included a mixed-methods approach with process evaluation and an experimental part. The process evaluation was conducted over time and guided by the MRC framework [5, 6], using descriptive data from managers and new graduates. The experimental part of the study were planned as a Randomized Controlled Trial (RCT) targeted newly graduated nurses with block-randomization to the intervention or control group. However, due to recruitment problems and thereof small sample sizes the study should be regarded as a pilot study.

### The intervention of peer learning in workplace introduction

A steering group consisting of the research group and managers was established to design the intervention. The intervention was tested and evaluated to identify how peer learning was received in the intended context [3]. The peer learning intervention involved a pair of new graduates undergoing workplace introduction together; they were scheduled on the same shifts, shared responsibility for a group of patients and were introduced by one preceptor. When using peer learning, the preceptor provided support and feedback when needed, but in contrast to traditional workplace introduction, he/she did not play an active role in nursing activities. The preceptors were registered nurses working on the ward and were chosen by the manager. Using a reflection card based on Gibbs’ reflection cycle [26] containing questions to ask themselves when reflecting, the pairs engaged in joint reflection. The first line manager selected one preceptor to be responsible for initiating and supporting the reflection. The intervention period was 3 months. The participating wards were offered information, and a leaflet describing the intervention was distributed to the managers, participants, preceptors, and co-workers to avoid multiple interpretations. The control group was introduced into the profession in the traditional manner, which involves “working” alongside an experienced nurse for a few weeks. Thus, one preceptor supervises one new graduate at a time.

### Settings and participants

#### Settings

The study was completed at three hospitals in central Sweden, including 22 eligible hospital wards. About 660 nurses graduated from the nearby university during the recruitment period, and 338 nurses were employed at the included wards.

#### First-line managers

The managers recruited and hired new graduates for the wards and were responsible for planning their workplace introduction. The inclusion criterion was that managers had received information about the study and agreed to participate in the study as soon as they hired a pair of newly graduated nurses who were to begin their introduction at the same time. Eight managers from seven hospital wards participated in group interviews during winter 2017–2018. They were divided into groups based on whether they had experience of the intervention (*n* = 5) or not (*n* = 3). The participants worked at two different hospitals.

#### New graduates

**Fig. 2:**
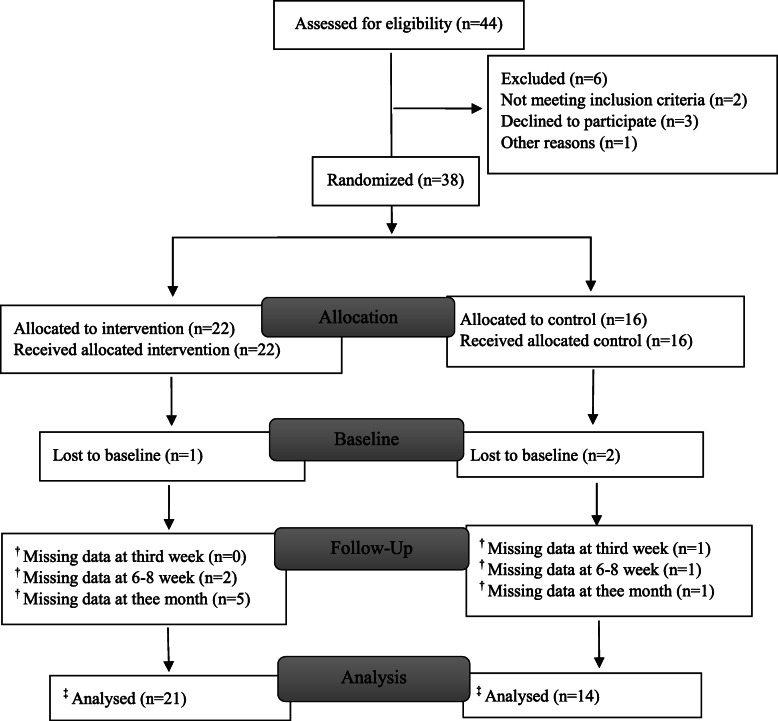
Flow chart of the respective group’s enrolment in the RCT-study. †The primary analysis was intention to treat and included all subjects as assigned with available baseline outcome data. ‡ Multiple imputation was used to address missing values

New graduates were invited to participate in the study between June 2015 and January 2018. The inclusion criterion was new graduates who, for the first time, were being introduced into the nursing profession and along with another new graduate starting workplace introduction at the same time. The procedure of the experimental part of the study involved: I) First-line managers informed the first author when they had a pair of new graduates starting introduction at the same time. II) The pairs were randomized in groups of ten. III) The manager informed the new graduates about their intended introduction. IV) The first author invited the pairs to participate in the study. The primary analysis was intention to treat and included all subjects assigned with baseline data. When the topic of investigation has not been studied previously – which is true of peer learning in workplace introduction of new graduates – the researcher may, according to Polit and Beck [27], estimate whether the expected effect is small, medium, or large. In nursing studies, small to medium effects are most common. Thus, the estimated sample size to be recruited was 76 participants in each group (*n* = 152), the goal being to achieve a medium effect size (0.50), a power of 0.80 [27] and a calculated attrition rate of about 20%. Due to recruitment problems only 44 new graduates were recruited for randomization, whereas 35 (80%) completed the baseline questionnaire. For information on enrolment in the respective groups, see Fig. 2.

### Data collection

#### Process evaluation – first-line managers

Group interviews with managers were used to study their experience of the peer learning intervention during new graduates’ work introduction. A semi-structured interview guide was used covering opening questions, introductory questions, transition questions, key questions and ending questions, inspired by Krueger [28], (Table 1). The interviews were conducted by the last author, lasted between 39 and 43 min and were tape-recorded, transcribed verbatim and checked for accuracy.

**Table 1:** Interview guide aims and questions inspired by Krueger [28]

	Aim	Questions
Opening questions	Get everyone to talk	Tell me about yourself? What kind of unit are you a first-line manager at? How common is the recruitment of new graduates at your unit?
Introductory questions	Introduce the topic	The introduction is a common concept of which you probably talk about when recruiting new graduates. What do you, as first-line manager wants the workplace introduction leading to or having effects on?
Transition question	Bring the discussion towards the key questions	Recalling the first time you heard about peer learning during workplace introduction. What were your thoughts?
Key questions		What do you, as a first-line manager consider of importance for the intervention to be successful? Do you experience any hindering factors? And if so, do you as a first-line manager have any facilities to manage these? Tell me about any positive outcomes you experienced on the new graduates due to the intervention (reflection included). Tell me about any negative outcomes you experienced on the new graduates due to the intervention (reflection included). What advice would you give if you were involved in developing the peer learning introduction?
Ending questions	Bring closure to the discussion and enabled participants to reflect on earlier comments.	What are your thoughts about content in the future introduction program? Is there anything you want to add that we have not talked about?

#### Process evaluation – new graduates in the intervention group

A checklist for fidelity was used to collect data, on seven occasions during the 3-month intervention period, from the 11 pairs in the intervention group.

In the end of the intervention period, the new graduates in the intervention group were asked to self-assess how important the joint reflection had been for their professional development with five response alternatives, from totally agree (1) to disagree (5).

#### The experimental part of the study – new graduates

To study the effects of the peer learning intervention, questionnaire data were collected on four occasions: at the beginning of the introduction period, after the introduction ended, 6 to 8 weeks- and 3 months after the new graduates started their introduction. The first author distributed the questionnaires to the participants’ workplace along with a stamped reply envelope. Ten validated instruments were used (Table 2). Two reminders were sent out by SMS.

**Table 2:** Tested outcome variables and instruments used with headlines reflecting program logic assumption (Fig. 1)

Outcome	Instruments used	Items
Learning & development		
Thriving	^a^Thriving scale [29]	The 11-item thriving scale measures two factors (vitality and learning) including five items each and a total scale. The scale has seven response options, where higher scores indicate greater levels of workplace thriving.
Belief in oneself		
Self-efficacy	^a^Nursing Self-Efficacy Scale (NSE) [30] A single item asking how prepared they were to cope with work as a nurse [31]	9-item scale with 11 response options, where 11 represents the most positive perception of nursing self-efficacy. This question had the same response categories as the NSE.
Psychological empowerment	^a^Spreitzer’s empowerment scale [32]	12-item scale, measuring four factors (meaning, competence, self-determination, and impact), and total scale. Seven response options, where higher scores the more positive perceptions of psychological empowerment.
Well-being		
Well-being	^a^WHO-5 Well Being Index (WHO-5) [33].	5-item scale with 6 response options where a score under 52 indicates poor well-being.
Job demands	^a^Specific job demands within the health care sector scale (SJDH – scale) [34].	The 15-item scale measuring four factors; pain and death; professional worries; patient and relative needs; threats and violence. Four response options, where higher scores indicate that they encountered various work-related elements to a higher extent.
Stress symptoms	^a^Psychosomatic health aspects scale [35].	11-item scale with 5 response options, where higher scores indicate a more desirable state.
Satisfaction		
Satisfaction with provided care	^a^The Nurse-specific Satisfaction with Care (NSC) [36].	9-item scale ranked with 7 response options, where higher scores indicate a higher level of satisfaction.
Job satisfaction	^a^the Job Satisfaction Questionnaire [37].	The 20-item scale measuring five factors (competence, emotion, autonomy, initiative, relation). Four response options, where high scores indicate high levels of job satisfaction.
	^a^The Brief Index of Affective Job Satisfaction (BIAJS) [38].	7-item scale with 5 response options scale, where higher score, indicates a higher level of satisfaction.

^a^Have been tested for validity and reliability with acceptable results

### Ethical considerations

Permission to conduct the study was obtained by the hospital managers and The Regional Ethical Review Board in Uppsala (2014/192), including an updated application concerning adding group interviews with first-line managers (2014/192/2). Participants received oral and written information about the study. The new graduates gave their written informed consent to participate in the study, and managers gave their verbal consent when participating in the group interviews. The participants were informed that their participation was voluntary, that they could withdraw from the study at any time, without any explanations or consequences, and that confidentiality was assured.

### Data analysis

#### Process evaluation – first-line managers

The audio-recorded group interviews with managers were analysed using qualitative content analysis inspired by Graneheim and Lundman [39]. The interviews were transcribed verbatim. Meaning units (sentences or paragraphs) were identified, coded and sorted in relation to the topics from the interview guide, i.e., factors facilitating and hindering intervention success as well as positive and negative outcomes for the new graduates due to the intervention. The codes within each topic were then grouped into sub-categories (Table 3). The analysis was conducted by the first author in dialogue with the last author. The identified codes, sub-categories and categories were discussed with all authors to ensure trustworthiness. The co-authors were senior researchers with considerable experience in conducting qualitative research.

**Table 3:** First-line managers’ experiences of the peer learning intervention, categories, and sub-categories

Advantages		Disadvantages	
Categories	Sub-categories	Categories	Sub-categories
Factors facilitating intervention success	Having support from researchers Giving support to new graduates and staff Being familiar with the peer learning model from nursing students	Factors hindering intervention success	A challenge to follow the intervention and study structure Being familiar with the peer learning model from nursing students
Positive peer learning outcomes	The pair learned from each other The pair supported each other Developed the wards view on new employees	Negative peer learning outcomes	Noticing problems when the pair was incompatible

#### Process evaluation – new graduates in the intervention group

Data from the checklist of intervention fidelity were transferred into a table consisting of essential parts of the intervention. The new graduates’ self-assessments of the importance of joint reflection are described in running text.

#### The experimental part of the study

Data from the questionnaire were analysed using IBM SPSS Statistics, version 24.0. Multiple imputations were used to address missing values and generalized estimating equations (GEE) models [40] for analyses of changes over time. To test if the effect of intervention changes over time an interaction term was included in all models. In the GEE, a sequential Bonferroni correction was applied (Additional file 1). Furthermore, to get effect size (ES) Cohen’s *d* we calculated change score within each group and then compared the groups’ change score using T-test. The point estimate for ES is from the five multiple imputations (the range) as SPSS do not calculate a pooled value for this. For Cohen’s *d*, the H_0_ is that *d* = 0, and a small, medium and large ES (or H_1_) are *d* = 0.20, 0.50 and 0.80 or more respectively [41].

### Trustworthiness

The last author conducted all group interviews, using a semi-structured interview guide to ensure they covered the same topics [27]. To reduce the risk of the researchers’ subjectivity affecting the data, the authors engaged in repeated discussions concerning interpretations and categorizations until consensus was reached. For the reader to assess the study’s credibility, participants’ characteristics, the analytical procedure and interview quotations are described [42].

Before starting data collection, a trial registration was conducted, a power analysis was estimated and the participants were randomized [27]. All scales used have previously documented validity and reliability. The research group translated the Brief Index of Affective Job Satisfaction (BIAJS) into Swedish, and a bilingual translator carried out a back-translation.

## Results

### Process evaluation – first-line managers

The managers interviewed were all females, had worked as manager between 2 months and 31 years and were responsible for hospital wards with 12 to 28 beds. Results from the analysis are presented below with excerpts from the interviews. In the related quotes, semi-colons (;) are used to mark when the participants are interacting and g1, g2, g3 are designate the different groups.

Factors facilitating and hindering intervention success:

Being randomized to the control group was experienced as a problem because the managers wanted to test the intervention. Furthermore, the new graduates did not begin their workplace introduction at the same time, which affected their opportunity to participate in the study.

*“Something else we’ve seen a lot in this project is that they didn’t start at the same time. That’s the dilemma”; “That’s really the problem”; “It’s not like you finish school on Friday and start on Monday. You take a couple weeks of vacation…” (g2)*

When a pair of new graduates were randomized to the intervention group, the managers were keen on following the study instructions, but were sometimes unsure about whether they had understood the study instructions. Receiving information and support from the researcher was described as important.

*“I asked myself several times – have I really understood this? Not just saying “yes” and then neglecting to get involved, that’s how I feel.” (g1)*

Some of the managers thought preparing for the intervention required some work, especially scheduling the reflection, whereas others thought there was no work worth mentioning. The managers expressed that scheduling the new graduates’ joint reflection was a change for the better, but they still experienced difficulties getting them to take the time to reflect. When the new graduates started their workplace introduction in pairs, the managers experienced practical issues if one of them was absent due to illness. The managers had encouraged the new graduates to complete the questionnaire, however the study participants found the repeated questionnaires too extensive and that same areas were repeated.

The managers described their own role in making the intervention work as giving the new graduates support and the choice of preceptor. Furthermore, they provided frameworks and information to help the staff work with and support the new graduates using peer learning. All of the managers, staff and new graduates had previous knowledge of peer learning, because the model have been used during nursing students’ clinical education.

*‘I think that you have to give information about what it is about so that the employees are understood.’ (g2)*

“*I can imagine since we started using this with students that it doesn’t feel unusual or strange.” (g1)*

Being familiar with the model was seen as positive, and structures used with students were also used for the new graduates’ introduction, although some skepticism was expressed. Sometimes familiarity could result in managers solving a problem like they did with students, but not in line with the intervention guideline.

“*…one of them took the initiative and the other didn’t dare. One was absent a lot at the beginning so it didn’t go so well.”; “A lack of balance.”; “Well, the pair wasn’t well balanced. Then we separated them for a while and then put them together. Then it was better. That’s what we do with students too if the two of them don’t work so well together.” (g1)*

The managers had experienced problems using peer learning, for example when the peers were incompatible. In all of the group interviews there were discussions about the advantage of the pair being compatible, as this results in the best possible outcome.

Positive and negative outcomes of the intervention:

The managers experienced that the pairs both learned to cooperate and learned together when cooperating.

*“They arrive at good things more quickly.” (g3)*

The pairs were perceived as independent; many of the nurse’s duties were managed within the pair. The managers experienced that the pairs supported and assisted each other, even after the intervention ended. This was described as generating feelings of safety for the new graduates, which the managers felt gave them the courage to ask questions and make demands.

*“They keep being a pair a long time, even if they don’t see each other privately or didn’t know each other while studying, they’re a pair who help each other a little extra. They’re stronger when there’s two of them, they dare to made demands, bring things up. They dare to speak out, “I’ve seen this,” they can discuss things they think aren’t right. But it’s probably easier when there’s two of them.” (g3)*

The managers felt it was advantageous for the new graduates to engage in joint reflection, as it allowed them to reflect on and share experiences.

*“This idea of time for reflection, and how important it is when you’re new. If a lot had happened, they could bring it up. It could be about medical things or conflicts or something, so those times for reflection, they were probably good.”; “It is beneficial, you can see that. It can serve them well.” (g1)*

The negative outcomes described by managers were predominantly seen in incompatible pairs. They described concerns that one in the pair could experience poorer development and poor self-confidence if the other was more resourceful.

Additionally, the managers reported that participating in the study improved the unit’s overall reception of new employees.

*“Personally, I think on my ward that everyone has grown thanks to this study… we’ve make pretty big changes.” (g1)*

### Process evaluation – new graduates in the intervention group

Regarding the checklist for intervention fidelity, the results showed that the participating pairs generally followed the intervention well including taking time for joint reflection (Table 4). However, during the intervention’s last 2 weeks, three of the pairs took time for joint reflection. Nine of the 16 new graduates who self-assessed the importance of the pairs’ joint reflection for their professional development considered it important, i.e., self-assessed 1 or 2 on the scale. Three new graduates self-assessed 3 on the scale.

**Table 4:** Overview of the intervention and intervention fidelity. The fidelity is based on the checklist for the intervention fidelity

	Intervention week 1, 2	Intervention week 3	Intervention week 4–6	Intervention week 4–12
The intervention	The pair worked the same shift, had joint responsibility for a group of patients and were introduced by one preceptor Daily joint scheduled reflection	The pair worked the same shift, but in contrast to the first 2 weeks they were solely responsible for a care team located next to each other Daily joint scheduled reflection.	Scheduled on the same shift twice a week	Joint reflection twice a week
^a^Intervention fidelity	Nine pairs of new graduates had joint responsibility for a care team for 2 weeks. Two pairs reported sickness in the pair. All pairs had daily joint reflection when working together.	One pair’s care team was not located next to each other One participant changed the scheme 2 days due to personal reasons Two pairs occasionally forgot or did not take the time to reflect every day	Ten pairs were scheduled the same shift twice a week. One pair had no shifts together.	Eight pairs had scheduled joint reflection twice a week between weeks 4 and 10. One pair reported they had joint reflection once a week between weeks 4 and 12. During the last 2 weeks three pairs had scheduled joint reflection twice a week. Reported reasons not to reflect were; high workload, sick leave, lack of time, one in the pair took a vacation

^a^The “Checklist for the intervention fidelity” was measured in all 11 pairs at weeks 1, 2, 3, 4–5, 7, 10 and 12

### The experimental part of the study

The results included 35 new graduates, 21 in the intervention group and 14 in the control group (Table 5). Due to the small sample sizes, only ES is reported here. The ES measured as Cohen’s *d* showed that 19 variables had a small ES, 7 variables ranged from small to medium effect (i.e. the point estimates from the 5 multiple imputations ranged from small to medium for that variable) and 3 had a medium effect size (Additional file 1). Due to small and medium ES in combination with small sample sizes, no conclusions can be drawn from the experimental part. Internal consistency was measured using Cronbach’s Alpha (Additional file 1).

**Table 5:** Characteristics of the new graduates in the intervention and control group

	Intervention group (*n* = 21)	Control group (*n* = 14)	Total (*n* = 35)
Age	21–47	22–39	21–47
Mean	27.9	26.7	27.4
Median	26	26	26
Gender			
Female	17	14	31
Male	4	0	4
^a^Worked in healthcare before the nursing program			
Yes	12	10	22
No	9	3	12
^a^Living arrangements			
Live alone	6	2	8
Live with parents	0	1	1
Live with partner/spouse	14	10	24
Live with friend	1	0	1
^a^Children			
Yes	9	5	14
No	12	8	20

^a^When sum up is less than 21, 14 or 35 there are internal missing data

## Discussion

The group interviews with managers revealed that there were practical problems when standardizing the intervention, but on the whole there were mostly positive descriptions of using peer learning during new graduates’ workplace introduction. Further, fidelity to the intervention was relatively good, and most followed the intervention components. However, during the latter part of the intervention period, the new graduates had problems taking time for joint reflection. The ES measured as Cohen’s d showed that 29 variables had a small or medium effect size with some mean differences in favor for the intervention group and some for the control group. As a consequence of recruitment problems (small sample sizes), it is difficult to draw conclusions concerning peer learning effects on new graduates. We can only add to the MRC statement that even at later stages in the development of complex interventions, process evaluations are important, as in present study, in considering the context of recruitment problems previously not identified.

Although there were problems in evaluating the effects of this peer learning intervention, the results from the interviews with the managers support the concept of two new graduates learning with and from each other during their workplace introduction. Managers’ experiences of peer learning have not been included in previous studies, although they are important individuals with responsibility for the work environment on their wards. In the study, the managers described themselves as having positive attitudes toward the intervention and experiencing several positive outcomes of using peer learning in new graduates’ introduction. The positive results described were that the pairs both learned while cooperating and learned how to cooperate. Furthermore, the managers found the pairs to be independent, and they noticed that the pairs supported and helped each other even after the intervention ended. This, in turn, was described as generating in the new graduates feelings of security and as giving the pairs the courage to ask questions and make demands. Similar positive outcomes, like those described by managers, have previously been reported by nursing students [14, 43], preceptors [44, 45] and new graduates [3, 46]. Interestingly, the managers’ descriptions of pairs giving each other the courage to ask questions and make demands are in line with data from interviews with new graduates using peer learning [3]. The managers’ experience of negative outcomes related to incompatible pairs has also been described by students [10, 16] and preceptors [44]. However, findings from a recent observational study showed that incompatible pairs of nursing students also practiced several competencies together, such as communication, reporting skills, organization of nursing care and leadership [16].

When measuring intervention fidelity, the results showed that participating pairs generally followed the intervention well. However, during the interventions last 2 weeks, only three pairs of new graduates took time for joint reflection. Reported reasons for not reflecting were, e.g., high workload and lack of time. Although the managers experienced the new graduates’ joint reflection as positive, they described difficulties getting the new graduates to prioritize that after the first 3 weeks. Furthermore, nine of 16 considered the joint reflection to be important to their professional development, and in previous peer learning studies peer reflection has been described as beneficial [3, 47]. The importance of managers’ involvement for intervention success was described in a previous study [25]. Moreover, an interview study on managers revealed the importance of reflection for new graduates’ learning and development [48]. One explanation for not taking time might be that new graduates do not want to be singled out for going away to reflect, as previous studies have reported on the importance of new graduates maintaining interpersonal relationships with colleagues [17, 49]. However, the first period of working in the nursing profession is described as associated with feelings of stress [50], where joint reflection might reasonably be supportive.

During the data collection in the experimental part of the study, it became obvious that the study had recruitment problems. The estimated sample size to be recruited was 76 pairs (152 participants). In the region, about 220 nurses graduated from the nearby university annually. During the recruitment of participants, i.e., between June 2015 and June 2018, 338 nurses were employed at the 22 eligible hospital wards. Of the estimated sample size of 152, 44 were recruited for randomization and 35 completed the baseline questionnaire. This was a surprise, and for this reason no concerns about the complexity of recruitment had surfaced in the feasibility study or in the steering group. In the earlier feasibility study [3], there was no problem recruiting pairs to the study, and there were no concerns about the complexity of recruitment in the steering group. In the group interviews, the managers reported not having the opportunity to participate in the study because the new graduates did not begin their workplace introduction at the same time and thus could not form pairs. This issue and being randomized to the control group were described as problems, because the managers were eager to test the intervention. Despite the great strength of an RCT, the study could have benefited from using a quasi-experimental design in which all new graduates that formed a pair would constitute the intervention group, and the comparison group would include new graduates starting their introduction alone. Considerations of alternatives to RCTs are also described in the updated version of the MRC framework. Furthermore, they suggest that even when a process evaluation has been conducted at the feasibility stage, another will usually be needed for the full trial, because new problems are likely to emerge when the intervention is tested in a larger more diverse sample [5, 6]. However, it is still important to publish “failures.” The World Health Organization (WHO) stated in a public disclosure [51] that about 50% of clinical trials go unreported, often because the results are negative. These unreported trial results leave an incomplete and potentially misleading picture of the risks and benefits of interventions.

### Limitations

Despite some useful results, the study has several limitations. The results must be interpreted with caution due to the small sample sizes. Owing to the low power, the study hypothesis can neither be accepted nor rejected. However, the MRC framework describes the importance of being aware that effects may be smaller or more variable, and response rates lower, when the intervention is tested in a larger sample, compared to results from a feasibility study [5]. The present study can be perceived as a pilot study. However, the intention was a full-scale RCT described in the trial registration, and the recruitment problems were unexpected.

The baseline questionnaire was completed at the end of the first week of introduction and might not be seen as a traditional baseline measurement, in that the new graduates had experienced their new workplace for a week. The managers reported that the study participants found the questionnaire to be too extensive which could have affected the response rate. Twelve weeks of intervention might be too long period, as most of the participants did not engage in reflection during the last 2 weeks.

## Conclusion

Process evaluations are important when developing and evaluating complex interventions. The study found positive experiences of and fidelity to the peer learning intervention. Regarding the experimental design, there were lessons learned, something also confirmed by the interviews with managers and stated in the MRC framework. Moreover, even after a feasibility study, when the process goes on to the next stage using an experimental study design, it is of great value to conduct a process evaluation to explore the interactions between context and study design that sometimes first emerge during the scale-up, which is also a result.

## Supplementary Information

**Additional file 1.**

## Data Availability

The datasets used and/or analysed during the current study are available from the corresponding author on reasonable request.
