# Supplementary material for: A peer learning intervention in workplace introduction - managers’ and new graduates’ perspectives
Source: BMC Nurs. 2022 Jan 4;21:12. doi: 10.1186/s12912-021-00791-0 (PMC8725265; doi:10.1186/s12912-021-00791-0)
Supplement: Supplementary file 1 — Additional file 1. [file 12912_2021_791_MOESM1_ESM.docx]

A PEER LEARNING INTERVENTION IN WORKPLACE INTRODUCTION - MANAGERS’ AND NEW GRADUATES’ PERSPECTIVES.

*Ylva Pålsson ^1, 2, *^, Maria Engström ^1, 2, 3^, Christine Leo Swenne ^2, 4^, Gunilla Mårtensson ^1, 2^*

| Additional file 1  New graduates’ self-rated performance over time in the intervention and control group as change over time between groups | | | | | | | | | |
| --- | --- | --- | --- | --- | --- | --- | --- | --- | --- |
|  |  |  | **GEE** | | | | | | **T-test** |
|  |  |  | ***Intervention Group (n=21)*** | ***Control Group (n=14)*** | ***Comparison of baseline measurement factors between groups*** | ***Change over time between groups*** | | |  |
|  |  |  |  |  |  |  |  |  |  |
| **Measurement factors** | **Baseline Cronbach´s α** | **Measurement** | **Mean** | **Mean** | ***p*-value ^†^** | **Measurement** | **Interaction coefficient** ^§^ | ***p*-value ^†^** | **Point estimate for ES,**  **Cohen´s *d*** *^‡^* |
| ***Learning & Development*** | | | | | | | | | |
| *Thriving Scale* | | | | | | | | | |
| Vitality | 0.84 | 1  2  3  4 | 5.3  5.3  5.1  5.1 | 5.6  5.6  5.6  5.4 | 0.411 | 1-2  1-3  1-4 | 0.233  0.084  0.265  0.080 | 0.759  0.261  0.773 | -0.06 to -0.12  -0.33 to -0.38  -0.04 to -0.21 |
| Learning | 0.65 | 1  2  3  4 | 6.3  6.4  6.1  6.1 | 6.4  6.4  6.5  6.6 | 0.492 | 1-2  1-3  1-4 | 0.129  - 0.075  0.252  0.373 | 0.734  0.193  0.062 | 0.06 to 0.17  -0.38 to -0.48  -0.51 to -0.67 |
| Total | 0.85 | 1  2  3  4 | 5.8  5.8  5.6  5.6 | 6.0  6.0  6.1  6.0 | 0.390 | 1-2  1-3  1-4 | 0.181  0.004  0.259  0.227 | 0.984  0.168  0.261 | -0.04 to 0.03  -0.40 to -0.45  -0.29 to -0.42 |
| ***Belief in oneself*** | | | | | | | | | |
| Self-efficacy  *Nursing Self Efficacy Scale (NSE)* | | | | | | | | | |
| Total | 0.88 | 1  2  3  4 | 8.9  8.8  8.6  8.9 | 8.7  8.5  8.5  8.8 | 0.642 | 1-2  1-3  1-4 | - 0.235  - 0.058  0.127  0.154 | 0.898  0.818  0.694 | -0.05 to 0.18  -0.31 to 0.07  -0.07 to -0.19 |
| *Prepared to cope with work as a nurse (single-item question)* | | | | | | | | | |
| Total |  | 1  2  3  4 | 4.7  5.0  5.2  5.1 | 5.1  4.8  5.5  5.2 | 0.317 | 1-2  1-3  1-4 | 0.438  - 0.633  - 0.129  - 0.381 | 0.134  0.752  0.473 | 0.46 to 0.68  -0.04 to 0.24  0.12 to 0.34 |
| Psychological empowerment  *Spreitzer’s empowerment scale* | | | | | | | | | |
| Meaning | 0.75 | 1  2  3  4 | 6.1  6.2  6.1  5.9 | 6.5  6.5  6.2  6.1 | 0.068 | 1-2  1-3  1-4 | 0.381  - 0.041  - 0.286  - 0.168 | 0.857  0.189  0.527 | - 1. to 0.08   0.32 to 0.53  0.14 to 0.31 |
| Competence | 0.87 | 1  2  3  4 | 4.8  4.9  5.1  5.2 | 5.4  5.2  5.4  5.5 | 0.134 | 1-2  1-3  1-4 | 0.540  - 0.229  - 0.240  - 0.289 | 0.465  0.434  0.401 | 0.19 to 0.27  0.19 to 0.28  0.12 to 0.33 |
| Self-determination | 0.88 | 1  2  3  4 | 3.8  3.9  4.1  4.1 | 4.4  4.4  4.6  4.4 | 0.077 | 1-2  1-3  1-4 | 0.690  - 0.187  - 0.176  - 0.467 | 0.654  0.597  0.285 | 0.07 to 0.22  0.06 to 0.23  0.22 to 0.39 |
| Impact | 0.89 | 1  2  3  4 | 3.6  3.7  3.9  4.0 | 4.4  4.3  4.5  4.3 | 0.055 | 1-2  1-3  1-4 | 0.786  - 0.208  - 0.167  - 0.467 | 0.535  0.688  0.266 | 0.17 to 0.27  0.09 to 0.20  0.31 to 0.42 |
| Total | 0.90 | 1  2  3  4 | 4.6  4.7  4.8  4.8 | 5.2  5.1  5.2  5.1 | ***0.024**** | 1-2  1-3  1-4 | 0.599  - 0.166  - 0.217  - 0.348 | 0.456  0.326  0.168 | 0.20 to 0.27  0.26 to 0.33  0.35 to 0.48 |
| *Well-being* | | | | | | | | | |
| *WHO-5 Well Being Index (WHO-5)* | | | | | | | | | |
| Total | 0.84 | 1  2  3  4 | 60.9  55.4  57.3  51.3 | 64.0  59.4  62.7  61.0 | 0.626 | 1-2  1-3  1-4 | 3.048  0.895  2.400  6.667 | 0.888  0.711  0.317 | -0.11 to 0.03  -0.02 to -0.22  -0.23 to -0.44 |
| Job demands  *Specific job demands within the health care sector scale (SJDH – scale)* | | | | | | | | | |
| Pain and death | 0.70 | 1  2  3  4 | 2.1  2.2  2.4  2.4 | 2.3  2.5  2.6  2.7 | 0.214 | 1-2  1-3  1-4 | 0.190  0.146  0.033  0.053 | 0.279  0.848  0.795 | -0.30 to -0.45  -0.01 to -0.13  -0.17 to 0.02 |
| Professional worries | 0.53 | 1  2  3  4 | 2.4  2.5  2.1  2.1 | 2.3  2.3  2.2  2.0 | 0.719 | 1-2  1-3  1-4 | - 0.083  - 0.112  0.164  - 0.071 | 0.540  0.414  0.748 | 0.16 to 0.29  -0.17 to -0.40  -0.05 to 0.20 |
| Patient and relative needs | 0.82 | 1  2  3  4 | 1.8  1.9  1.9  2.2 | 2.0  1.9  2.1  2.1 | 0.239 | 1-2  1-3  1-4 | 0.236  - 0.151  0.024  - 0.396 | 0.502  0.921  0.089 | 0.20 to 0.32  -0.07 to 0.05  0.51 to 0.68 |
| Threats and violence | 0.77 | 1  2  3  4 | 1.1  1.3  1.3  1.7 | 1.5  1.8  1.5  1.6 | ***0.011**** | 1-2  1-3  1-4 | 0.349  0.200  - 0.144  - 0.405 | 0.233  0.338  0.114 | -0-44 to -0.49  0.24 to 0.44  0.42 to 0.76 |
| Stress symptoms  *Psychosomatic health aspects scale* | | | | | | | | | |
| Total | 0.78 | 1  2  3  4 | 1.9  1.9  1.7  1.6 | 2.2  2.0  1.9  1.9 | 0.257 | 1-2  1-3  1-4 | 0.233  - 0.153  - 0.075  0.061 | 0.413  0.664  0.768 | 0.22 to 0.30  0.06 to 0.20  -0.03 to -0.14 |
| *Satisfaction* | | | | | | | | | |
| Satisfaction with provided care  *The Nurse-specific Satisfaction with Care (NSC)* | | | | | | | | | |
| Total | 0.88 | 1  2  3  4 | 5.1  5.1  5.1  5.3 | 5.2  5.4  5.4  5.5 | 0.519 | 1-2  1-3  1-4 | 0.185  0.122  0.174  - 0.033 | 0.618  0.432  0.904 | -0.14 to -0.20  -0.19 to -0.31  -0.02 to 0.12 |
| Job satisfaction  *The Job Satisfaction Questionnaire* | | | | | | | | | |
| Competence | 0.90 | 1  2  3  4 | 2.4  2.5  2.3  2.4 | 2.7  2.7  2.5  2.5 | 0.077 | 1-2  1-3  1-4 | 0.257  - 0.150  - 0.035  - 0.110 | 0.274  0.858  0.553 | 0.27 to 0.39  0.02 to 0.08  0.09 to 0.35 |
| Emotion | 0.89 | 1  2  3  4 | 2.0  2.2  2.0  2.0 | 2.5  2.3  2.4  2.4 | ***0.021**** | 1-2  1-3  1-4 | 0.410  - 0.275  0.050  - 0.011 | 0.172  0.814  0.955 | 0.36 to 0.52  -0.03 to -0.14  0.00 to 0.05 |
| Autonomy | 0.73 | 1  2  3  4 | 1.6  1.6  1.5  1.5 | 1.7  1.5  1.6  1.6 | 0.460 | 1-2  1-3  1-4 | 0.135  - 0.213  0.054  0.076 | 0.322  0.790  0.758 | 0.26 to 0.37  -0.16 to 0.00  0.03 to 0.22 |
| Initiative | 0.86 | 1  2  3  4 | 1.9  2.1  1.9  2.0 | 2.3  2.1  2.2  2.1 | 0.062 | 1-2  1-3  1-4 | 0.377  - 0.345  - 0.064  - 0.292 | ***0.033****  0.791  0.180 | 0.61 to 0.71  -0.02 to 0.18  0.31 to 0.55 |
| Relation | 0.87 | 1  2  3  4 | 2.3  2.4  2.1  2.2 | 2.3  2.4  2.4  2.5 | 0.846 | 1-2  1-3  1-4 | 0.040  - 0.016  0.330  0.294 | 0.940  0.182  0.235 | -0.05 to 0.09  -0.39 to -0.42  -0.30 to -0.47 |
| *The Brief Index of Affective Job Satisfaction (BIAJS)* | | | | | | | | | |
| Total | 0.74 | 1  2  3  4 | 3.9  3.6  3.6  3.4 | 3.9  4.0  4.0  3.9 | 0.879 | 1-2  1-3  1-4 | 0.032  0.330  0.379  0.461 | 0.157  0.153  0.082 | -0.31 to -0.69  -0.29 to -0.69  -0.46 to -0.83 |
| *† Generalized estimating equations (GEE) models whereat* *a sequential Bonferroni correction was applied*  *‡ T-test. To get effect size (ES) Cohen’s d we calculated change score within each group and then compared the groups’ change score using T-test. The point estimate for ES is from the five multiple imputations (the range) as SPSS do not calculate a pooled value for this*  *§ The interaction coefficient quantifies how the effect of intervention on the variables changes by each occasion i.e. 1-2, 1-3, and 1-4*  ** The significance level is p≤ 0.05, and statistically significant results are marked with boldface text.*  *For all scales and factors except job demands, a higher score is a desired value. For job demands, a higher value indicate that they encountered various work-related elements to a higher extent* | | | | | | | | | |
